# The Ruts of Life

**Published:** 1871-07

**Authors:** 


### THE RUTS OF LIFE.

GET out of them, if you wish to live long, if you wish to avoid the lunatic asylum, if you wish to escape suicide or a miser's death. Men and women must have recreation, must have amusement, must have diversion. It is wholesome for the mind to break away from its daily vocation or employment every night. The man who goes from his counting-house or his workshop at the close of the day and does not leave it behind him, but sits at the family table in moodiness, brooding over past occurrences, weighing probabilities, casting conjectures, laying plans, and when the meal is over sits thinking, thinking, thinking by the hour, and goes to bed to toss and tumble and worry, cannot live long; the brain or the heart must give way, and he will drop dead in the street, as many a business New Yorker has done within a few years past.

In the Island of Cuba, the wagon roads lead over hills made of limestone ; the wheels have run in the same track for generations, and have so worn into the solid stone that the hubs scrape the surface, and there is no getting out of the rut until the bottom of the hill is reached. So in the lives of many the mind, under the influence of worldly care, gets to run in a particular track ; in other cases, the occupations are of such an insufferable sameness from one year's end to another, that its workings become mechanical, and out of these lines they cannot work at all; hence the stupidity of such a large portion of the farming population of all countries; the peasants of England and Ireland and France, and Germany and Russia as well.

More farmers' wives and daughters go crazy, out of one thousand, than of any other class, simply because of the one same routine of drudgery, — of cooking, washing, cleaning, from morning to night, from one year's end to another; even the Sabbath day making but little change, and that change only the result of the extra drudge of Saturday.

And our wives, in large towns and cities, sweep and dust and arrange, and wash and sew and provide, in one incessant round, summer and winter. No wonder they grow thin and careworn, and weak and nervous. Get out of the ruts, all of you; pay a neighborly visit three nights in the week; or for two afternoons let there be a "let up" in the way of a drive to the Central Park, a visit to the "village," an excursion on the river or in the cars, a picnic, a celebration, but best of all, in city or country, a horseback ride of an hour or two, "there and back;" what an appetite it gives; and the weariness, what a delicious sleep follows!

Get out of the rut, reader, two or three hours a week, and there will be no time lost by it in the long run; for it gives activity to the moral nature; it cultivates the affections; it wakes up observation; it exercises comparison; it gives breadth of view on all subjects; it makes a man more manly; it makes a woman more womanly; and in countless cases it would save from the madhouse!

